# Eosinophils Target Therapy for Severe Asthma: Critical Points

**DOI:** 10.1155/2018/7582057

**Published:** 2018-10-25

**Authors:** L. Brussino, E. Heffler, C. Bucca, S. Nicola, G. Rolla

**Affiliations:** ^1^Department of Medical Science, University of Torino, Italy; ^2^Allergy and Clinical Immunology Unit, AO Ordine Mauriziano Umberto I, Torino, Italy; ^3^Personalized Medicine, Asthma and Allergy, Humanitas Research Hospital, Rozzano, Italy; ^4^Department of Biomedical Sciences, Humanitas University, Rozzano, Italy; ^5^Azienda Ospedaliero Universitaria Città della Salute e della Scienza, S.C. Pneumologia U, 10126 Torino, Italy

## Abstract

Asthma is a chronic and heterogeneous disease, which is defined as severe disease whenever it requires treatment with a high dose of inhaled corticosteroids plus a second controller and/or systemic corticosteroids to prevent it from becoming ‘‘uncontrolled” or if it remains ‘‘uncontrolled” despite this therapy. Severe asthma is a heterogeneous condition consisting of phenotypes such as eosinophilic asthma, which is characterized by sputum eosinophilia, associated with mild to moderate increase in blood eosinophil count, frequently adult-onset, and associated with chronic rhinosinusitis with nasal polyps in half of the cases. Eosinophilic asthma is driven by T2 inflammation, characterized, among the others, by interleukin-5 production. IL-5 plays a key role in the differentiation, survival, migration, and activation of eosinophils, and it has become an appealing therapeutic target for eosinophilic asthma. In recent years two monoclonal antibodies (mepolizumab and reslizumab) directed against IL-5 and one monoclonal antibody directed against the alpha-subunit of the IL-5 receptor (benralizumab) have been developed. All these IL-5 target drugs have been shown to reduce the number of exacerbation in patients with severe asthma selected on the basis of peripheral blood eosinophil count. There are still a number of unresolved issues related to the anti-IL5 strategy in eosinophilic asthma, which are here reviewed. These issues include the effects of such therapy on airway obstruction and asthmatic symptoms, the level of baseline eosinophils that predicts a response to treatment, the relationship between blood and airway eosinophilia, and, perhaps most importantly, how to elucidate the pathogenetic role played by eosinophils in the individual patient with severe eosinophilic asthma.

## 1. Introduction

Asthma is a chronic disease characterized by different clinical presentations, comorbidities, and outcomes, affecting an estimated 300 million people worldwide, of all ages, who usually need many specialists in order to be well managed [1–5]. Although asthma is generally mild and well controlled, the severe form, which represents at most 10% of asthmatic patients, can be refractory to conventional therapies, such as inhaled corticosteroids (ICSs), inhaled bronchodilators, and oral leukotriene modifiers [6, 7].

The outcome of asthma therapy becomes very important in terms of public health, social impact, and quality of life, particularly for those people suffering from severe asthma. It is therefore becoming more and more important to identify patients' phenotypes and to target precise molecules to obtain a good asthma control.

## 2. Asthma Phenotypes and Endotypes

Asthma can be classified into different phenotypes, according to its clinical presentation, concomitant comorbidities such as nasal polyposis or obesity, identifiable triggers, including allergen or aspirin sensitivity and response to therapy. Phenotypes, as measurable and observable features of asthma, are also available in defining eosinophilic and noneosinophilic asthma. In fact, the lack of knowledge of pathogenesis underlying each different phenotype represents a limit in understanding the mechanisms of Asthma subgroups and in disease management. Recently it has been proposed that the definition of endotype represents a specific biological mechanism which underlies a given phenotype.

The identification of different endotypes provides a contribution to lead novel treatments, such as biologic therapies to target specific inflammatory mediators (e.g., IL-5) [8, 9].

The two-main recognized asthma endotypes are based on high or low T-helper 2 (TH2) cell airway inflammation. Considering also type 2 innate lymphoid cells (ILC2), which are outside the originally described Th2 cell population but producing the same cytokines, (T2)-high or (T2)-low has emerged as a more appropriate and inclusive term for defining asthma endotypes.

The pathophysiology of T2 low asthma is not completely understood, but it is thought that it could be characterized by neutrophilic inflammation, suggesting a TH1 and/or TH17 cells activation.

On the other hand, in T2 high asthma, overproduction of eosinophils, driven by an overproduction of type 2 cytokines from T-helper 2 and innate lymphoid cells, is commonly found in many patients, and it correlates with more severe disease, with airway dysfunction [10].

## 3. Eosinophilic Asthma and IL5

In more than 50% of patients affected by severe eosinophilic asthma (SEA), both blood and sputum eosinophilia are associated with worse disease control and prognosis [11]. In addition, blood eosinophilia often reflects asthma severity [12] and the relationship between the reduction in sputum eosinophils and the reduction of exacerbations after ICS therapy is well recognized [13].

Interleukin 5 (IL-5) is a cytokine produced by limited types of cells, such as CD4+ T cells, innate lymphocytes type 2 (ILC-2), mast cells, and eosinophils, which are all involved in the airway inflammation of asthma. Whatever the source, IL-5 plays a major role in the differentiation, survival, migration, and activation of eosinophils. This is the reason why IL-5 represents an appealing therapeutic target for hypereosinophilic conditions.

## 4. Anti-IL5 Strategy in Eosinophilic Asthma

At the beginning of 2000s, the therapeutic role of IL-5 antagonists in asthma was postulated following the observation in rats of the eosinophils reduction in BAL and lung tissue and reduction of airway hyperresponsiveness after treatment with anti-IL5 monoclonal Antibodies (mAbs) intranasally, intravenously, or intraperitoneally, suggesting a good outcome also in treatment of human asthma [14].

Leckie et al. analyzed the effects of mepolizumab, an anti-IL5 monoclonal antibody, in 24 patients with mild asthma, observing a reduction of eosinophils in sputum and blood after allergen challenge, but they did not find a decrease in airway hyperresponsiveness to histamine or in late reaction after allergen challenge [15]. A few years later, it was observed that mepolizumab induced reduction in blood eosinophilia and a slight improvement in FEV1 in asthmatic patients taking high doses of ICS and/or oral corticosteroids, without significant changes in other clinical outcomes [16].

The efficacy of mepolizumab in patients with eosinophilic asthma has been preliminary reported in in 2009 in 2 randomized double-blind, placebo controlled studies. In the first one, Haldar demonstrated the reduction of exacerbations and the improvement in AQLQ scores in 29 patients with refractory eosinophilic asthma. The second study by Nair and Coll reported the reduction of eosinophils in blood and sputum, as well as prednisone sparing in 9 patients who had asthma with sputum eosinophilia despite prednisone treatment. In both studies patients received 750 mg intravenously of mepolizumab for 12 and 5 months, respectively [17, 18].

Later, in 2012 another study reported the efficacy of mepolizumab in a group of patients affected by eosinophilic asthma [19]. These observations placed the basis for the selection of patients based on the disease phenotype to achieve a tailored therapy. Furthermore, the knowledge of eosinophils involvement in asthma and the potential to block IL-5 stimulated other research studies to better identify the field of application of the new anti-IL5 mAbs [20].

In the last two years two similar biologics therapies targeting IL-5, mepolizumab and reslizumab, have been approved, as well as anti-IL-5 alpha receptor, benralizumab. These agents can be used as add-on therapy in subjects with an eosinophilic asthma phenotype, poorly controlled with standard therapy. Mepolizumab and reslizumab both target and bind to IL-5 directly, whereas benralizumab targets the IL-5 receptor alpha subunit. [21, 22].

The primary outcome in mepolizumab registration studies was the reduction of annual frequency of significant asthma exacerbations, which was defined as worsening of asthma which needed to be treated with systemic glucocorticoids for at least 3 days or when the patient visited an emergency department or was hospitalized. Secondary endpoints were the effects of treatment on blood eosinophil counts, asthma control evaluated by ACQ-5 score, asthma-related quality of life, and forced expiratory volume in 1 s (FEV1) [19]. Due to the hierarchical gatekeeping in the study design, the secondary endpoints were not analyzed in the registration study, not having reached the significant difference in reduction of exacerbation requiring ED admission between iv mepolizumab and placebo [23].

On the other side, primary outcome for reslizumab registration studies was the change from baseline in prebronchodilator FEV1 over 16 weeks. Secondary endpoints included ACQ scores, FVC, forced expiratory flow at 25% to 75% of FVC (FEF25%-75%), patient-reported control of asthma symptoms, short-acting *β*-agonist (SABA) use in the three days before the visit, blood eosinophil levels, and safety [24]. All the secondary endpoints were reached except for ACQ, which did not show any difference between reslizumab and placebo.

Lastly, the primary outcome in benralizumab registration studies was annual exacerbation rate ratio versus placebo for patients receiving high-dosage ICS plus LABA with baseline blood eosinophils 300 cells per *μ*L or greater (intention-to-treat analysis), while secondary efficacy endpoints were prebronchodilator FEV1 and total asthma symptom score for patients receiving high-dose inhaled corticosteroids plus LABA with baseline blood eosinophils 300 cells per *μ*L or greater. Additional secondary endpoints were time to first asthma exacerbation, annual rate of asthma exacerbations associated with emergency department visit, urgent care visit, or admission to hospital (defined as an admission to an inpatient facility and/or evaluation and treatment in a health-care facility for 24 hours or longer), postbronchodilator FEV1, ACQ-6 score, and AQLQ(S)+12 score. The annual rate of asthma exacerbations requiring an ED admission did not differ between the benralizumab and placebo, and benralizumab treatment did not alter the time to first asthma exacerbation requiring an emergency department visit or admission to hospital [25].

Analyzing the efficacy studies of the three Anti-IL5 mAbs, it is important to focus on the primary outcome, which is, for mepolizumab and benralizumab [19, 21], the reduction in annual asthma exacerbation numbers and, limited to reslizumab, the improvement in lung function test [24].

The study design for mepolizumab considered only patients with at least two exacerbations in the last year, showing a significant reduction rate in exacerbation of 53% for the group receiving subcutaneous mepolizumab [19], with an exacerbation rate of 0.93/year.

A secondary (post hoc) analysis of data from two randomized, double-blind, placebo-controlled studies of at least 32 weeks duration, DREAM and MENSA, was performed to evaluate the relationship between baseline eosinophil counts and efficacy of Mepolizumab, stratifying patients by different baseline eosinophil thresholds (count and ranges) in the blood, specifically baseline ≥150, ≥300, ≥400, and ≥500 cells per *μ*L, and baseline blood eosinophil ranges (<150 cells per *μ*L, ≥150 cells per *μ*L to <300 cells per *μ*L, ≥300 cells per *μ*L to <500 cells per *μ*L, and ≥500 cells per *μ*L).

It was observed that the exacerbation rate reduction with mepolizumab versus placebo increased progressively from 52% in patients with a baseline peripheral eosinophil count of at least 150 cells per *μ*L to 70% in those with a baseline count of at least 500 cells per *μ*L. When the baseline eosinophil count was less than 150 cells per *μ*L, the efficacy of mepolizumab was clearly reduced [26].

## 5. Unresolved Issues Related to the Anti-IL5 Therapy in Eosinophilic Asthma

### 5.1. Annual Exacerbation Rate Reduction

One important question is the clinical meaning of 50% reduction of annual exacerbation rate and the primary outcome of mepolizumab studies, in the patients who report just two exacerbation/year.

Even if exacerbation as defined in clinical trials is an “all-or-nothing” parameter, it is well recognized that patients with high symptom burden have more frequent exacerbations. The decrease of annual exacerbations per se does not automatically imply a comparable decrease of daily asthmatic symptoms, even if a 50% decrease in asthmatic symptoms has been reported in the same registration studies.

Other clinical endpoints, in addition to annual exacerbation rate, as the need of frequent oral corticosteroid use, asthmatic symptoms and quality of life, should be taken into account by clinicians who consider anti-IL-5 therapy for their patients with severe asthma.

### 5.2. FeNO as a Marker of T2 Inflammation

Outcomes which have been explored in secondary registration studies were the reduction in blood eosinophil count, the improvement in quality-of-life, the increase of forced expiratory flows, and the reduction in using of SABA. Markers of tissue eosinophilia which have been investigated in these studies were the sputum eosinophilia and, in one study only, the change of exhaled nitric oxide (FeNO) after mepolizumab therapy.

FeNO measurement is esteemed to be particularly useful to identify a high T2 state. The official ATS clinical practice guideline recommends that high FeNO > 50 ppb in adults and > 35 ppb in children can be used to indicate eosinophilic inflammation [27] in subjects, not in steroid therapy.

However, in spite of the expected correlation between the FeNO values and the number of eosinophils in the sputum of patients with eosinophilic asthma, in clinical setting a discrepancy is often observed between the two aforementioned values: high FeNO values may be sometimes observed in patients with normal sputum eosinophils count and* vice versa*.

Mepolizumab has been shown to decrease consistently blood and sputum eosinophil counts in patients with eosinophilic asthma, with no effect on FeNO levels [17].

A possible explanation of the absence of efficacy of mepolizumab on FeNO is that the molecular pathways that lead to an increase in FeNO are different from those that underlie the recruitment and activation of eosinophils, as the FeNO is mainly related to the pathways involved in T2 mediated asthma, while peripheral eosinophilia in asthmatic patients depends also upon the activity of lymphoid cells (ILC2) type 2 [28].

Recent evidence shows indeed that, in atopic asthma, the production of FeNO is stimulated by proinflammatory T2-cytokines, other than IL-5, such as IL-4 and IL-13, making NO a biomarker of T2-driven inflammation [29], which is not susceptible to the action of the anti-IL5 mAbs [30].

### 5.3. Eosinophil Count for the Assessment of Anti-IL5 Efficacy

Peripheral blood and sputum eosinophil counts have been shown to be consistently decreased by anti-IL5 drugs in all the three registration studies [23–25].

It is well known that both blood and pulmonary eosinophils are increased in patients with eosinophilic asthma. Whether peripheral blood eosinophils mirror bronchial tissue eosinophilia is not known. Also how important is the pathogenetic role played by eosinophils in asthma is not completely known. Bronchial eosinophilia may persist even when peripheral blood eosinophil count has been reduced by anti-IL-5 treatment [31].

Even the reduction of bone-marrow eosinophils, obtained by benralizumab treatment, was not able to abolish eosinophils infiltration in bronchial biopsies [13] or ECP levels in the sputum [32]. This observation suggests the important role of local mechanisms and/or of other cytokines in promoting eosinophils priming, recruitment, activation, and survival in the tissues. Nevertheless, as the source of eosinophil in the tissue is from the blood it is conceivable as a cumulative benefit on tissue eosinofil level with persistent blockade of blood eosinophils, but the duration of blockade required for measurable benefit on tissue eosinophils has not been evaluated.

The best predictor of response to ICS/OCS in patients with airway diseases, not only asthma, but also COPD and chronic cough of eosinophilic bronchitis, is the presence of eosinophils into the bronchial tissue, which is also predictive of response to therapies that indirectly target eosinophils such as anti-(IL-5) monoclonal antibodies [33, 34]. Whether blood or sputum eosinophils levels are the best predictor of therapy response need to be assessed in specific studies.

Any asthma therapy which had eosinophils as target will be much more effective the more it decreases airway eosinophils and the more airway eosinophils are primary players of airway inflammation. Unfortunately, markers of airways eosinophils activation are not currently available, and this is probably a great limitation to identify the asthmatic patients who could benefit more from anti-IL5 therapies.

Free eosinophil granules (FEGs), released after eosinophils' lysis, are detectable in sputum of patients with uncontrolled and severe asthma and the measure of sputum FEGs could be a new marker of eosinophilic airway inflammation [35].

FEGs contain toxic proteins which are responsible for bronchial epithelial damage, and their presence in the sputum is the consequence of eosinophils degranulation, which is an important mechanism of tissue damage driven by eosinophils [33, 36]. Moreover, the release of eosinophils' peroxidase (EPX) has been related to local airway autoimmunity, following the production of anti-EPX antibodies. This autoimmune mechanism has been related not only to failure of mepolizumab therapy but even to worsening of asthma control in some patients with severe eosinophilic asthma [37, 38] who receive the standard dose of mepolizumab (100 mg s.c.). The same patients had been shown to respond to higher dose or to i.v. reslizumab [18, 23].

It has been suggested that lower doses of anti-IL-5 drugs might not neutralize completely IL-5, which could still be able to promote local airway eosinophilia, in spite of the normalization of the blood eosinophil count [39, 40].

Another possibility is that a lower dose of anti-IL5 Mab could drive airway eosinophilia through the production of immune-complex and/or complement activation [38]. Such immune complexes could act as depot of IL5, leading to an increase in biological activity of the bound IL-5 [41]. This is a theoretical risk that has not been corroborated by clinical studies.

It has been recently demonstrated [42] that levels of Ig-bound IL-5 in the sputum of mepolizumab nonresponder patients was associated with increase in sputum eosinophils count.

In conclusion, anti-IL-5 treatment is a novel therapeutic strategy which may offer many clinical benefits to an asthmatic patients, selected on the basis of recurrent asthmatic exacerbation due to eosinophilic airway inflammation. Certainly, such a strategy is not an option for patients suffering from moderate persistent asthma, particularly if they do not need frequent oral corticosteroid courses to obtain asthma control. On the other hand, patients who, despite receiving systemic glucocorticoids, had peripheral blood eosinophil count well above 150 cells/mcL and frequent asthma exacerbations would experience better control of asthma symptoms along with reduced exacerbation rates [23, 43, 44].

Furthermore, the glucocorticoid-sparing effect of anti-IL-5 therapy [44] may prevent the serious, often irreversible adverse effects of glucocorticoids.

## References

[1] Corren J. (2013). Asthma phenotypes and endotypes: an evolving paradigm for classification. *Discovery Medicine*.

[2] Agusti A., Bel E., Thomas M. (2016). Treatable traits: toward precision medicine of chronic airway diseases. *European Respiratory Journal*.

[3] Chung K. F. (2015). Targeting the interleukin pathway in the treatment of asthma. *The Lancet*.

[4] Chung K. F., Wenzel S. E., Brozek J. L. (2014). International ERS/ATS guidelines on definition, evaluation and treatment of severe asthma. *European Respiratory Journal*.

[5] Masoli M., Fabian D., Holt S., Beasley R. (2004). The global burden of asthma: executive summary of the GINA Dissemination Committee Report. *Allergy: European Journal of Allergy and Clinical Immunology*.

[6] Barnes P. J., Adcock I. M. (2009). Glucocorticoid resistance in inflammatory diseases. *The Lancet*.

[7] Pepper A. N., Renz H., Casale T. B., Garn H. (2017). Biologic Therapy and Novel Molecular Targets of Severe Asthma. *The Journal of Allergy and Clinical Immunology: In Practice*.

[8] Lötvall J., Akdis C. A., Bacharier L. B. (2011). Asthma endotypes: a new approach to classification of disease entities within the asthma syndrome. *The Journal of Allergy and Clinical Immunology*.

[9] Carr T. F., Zeki A. A., Kraft M. (2018). Eosinophilic and noneosinophilic asthma. *American Journal of Respiratory and Critical Care Medicine*.

[10] Jared Darveaux J., Busse W. W. (2015). Biologics in Asthma – The Next Step Towards Personalized Treatment. *Journal of Allergy and Clinical Immunology: In Practice*.

[11] Buhl R., Humbert M., Bjermer L. (2017). Severe eosinophilic asthma: a roadmap to consensus. *European Respiratory Journal*.

[12] Busse W., Spector S., Rosén K., Wang Y., Alpan O. (2013). High eosinophil count: A potential biomarker for assessing successful omalizumab treatment effects. *The Journal of Allergy and Clinical Immunology*.

[13] Laviolette M., Gossage D. L., Gauvreau G. (2013). Effects of benralizumab on airway eosinophils in asthmatic patients with sputum eosinophilia. *The Journal of Allergy and Clinical Immunology*.

[14] Shardonofsky F. R., Venzor J., Barrios R., Leong K.-P., Huston D. P. (1999). Therapeutic efficacy of an anti-IL-5 monoclonal antibody delivered into the respiratory tract in a murine model of asthma. *The Journal of Allergy and Clinical Immunology*.

[15] Leckie M. J., Ten Brinke A., Khan J. (2000). Effects of an interleukin-5 blocking monoclonal antibody on eosinophils, airway hyper-responsiveness, and the late asthmatic response. *The Lancet*.

[16] Kips J. C., O'Connor B. J., Langley S. J. (2003). Effect of SCH55700, a humanized anti-human interleukin-5 antibody, in severe persistent asthma: A pilot study. *American Journal of Respiratory and Critical Care Medicine*.

[17] Haldar P., Brightling C. E., Hargadon B. (2009). Mepolizumab and exacerbations of refractory eosinophilic asthma. *The New England Journal of Medicine*.

[18] Nair P., Pizzichini M. M. M., Kjarsgaard M. (2009). Mepolizumab for prednisone-dependent asthma with sputum eosinophilia. *The New England Journal of Medicine*.

[19] Nair P. (2014). Anti-interleukin-5 monoclonal antibody to treat severe eosinophilic asthma. *The New England Journal of Medicine*.

[20] Flood-Page P., Swenson C., Faiferman I. (2007). A study to evaluate safety and efficacy of mepolizumab in patients with moderate persistent asthma. *American Journal of Respiratory and Critical Care Medicine*.

[21] Khorasanizadeh M., Eskian M., Assa’ad A. H., Camargo C. A., Rezaei N. (2016). Efficacy and Safety of Benralizumab, a Monoclonal Antibody against IL-5R*α*, in Uncontrolled Eosinophilic Asthma. *International Reviews of Immunology*.

[22] Giannetti M. P., Cardet J. C. (2016). Interleukin-5 antagonists usher in a new generation of asthma therapy. *Current Allergy and Asthma Reports*.

[23] Ortega H. G., Liu M. C., Pavord I. D. (2014). Mepolizumab treatment in patients with severe eosinophilic asthma. *The New England Journal of Medicine*.

[24] Bjermer L., Lemiere C., Maspero J., Weiss S., Zangrilli J., Germinaro M. (2016). Reslizumab for Inadequately Controlled Asthma With Elevated Blood Eosinophil Levels: A Randomized Phase 3 Study. *CHEST*.

[25] FitzGerald J. M., Bleecker E. R., Nair P. (2016). Benralizumab, an anti-interleukin-5 receptor *α* monoclonal antibody, as add-on treatment for patients with severe, uncontrolled, eosinophilic asthma (CALIMA): a randomised, double-blind, placebo-controlled phase 3 trial. *The Lancet*.

[26] Ortega H. G., Yancey S. W., Mayer B. (2016). Severe eosinophilic asthma treated with mepolizumab stratified by baseline eosinophil thresholds: a secondary analysis of the DREAM and MENSA studies. *The Lancet Respiratory Medicine*.

[27] Dweik R. A., Boggs P. B., Erzurum S. C. (2011). An official ATS clinical practice guideline: interpretation of exhaled nitric oxide levels (FENO) for clinical applications. *American Journal of Respiratory and Critical Care Medicine*.

[28] Coumou H., Bel E. H. (2016). Improving the diagnosis of eosinophilic asthma. *Expert Review of Respiratory Medicine*.

[29] Bjermer L., Alving K., Diamant Z. (2014). Current evidence and future research needs for FeNO measurement in respiratory diseases. *Respiratory Medicine*.

[30] Crespo A., Giner J., Torrejón M. (2016). Clinical and inflammatory features of asthma with dissociation between fractional exhaled nitric oxide and eosinophils in induced sputum. *Journal of Asthma & Allergy Educators*.

[31] Papathanassiou E., Loukides S., Bakakos P. (2016). Severe asthma: anti-IgE or anti-IL-5?. *European Clinical Respiratory Journal*.

[32] Busse W. W., Katial R., Gossage D. (2010). Safety profile, pharmacokinetics, and biologic activity of MEDI-563, an anti-IL-5 receptor *α* antibody, in a phase I study of subjects with mild asthma. *The Journal of Allergy and Clinical Immunology*.

[33] Nair P., Ochkur S. I., Protheroe C. (2013). Eosinophil peroxidase in sputum represents a unique biomarker of airway eosinophilia. *Allergy: European Journal of Allergy and Clinical Immunology*.

[34] Mukherjee M., Sehmi R., Nair P. (2014). Anti-IL5 therapy for asthma and beyond. *World Allergy Organization Journal*.

[35] Persson C. (2014). Primary lysis of eosinophils in severe desquamative asthma. *Clinical & Experimental Allergy*.

[36] Mukherjee M., Nair P. (2015). Blood or sputum eosinophils to guide asthma therapy?. *The Lancet Respiratory Medicine*.

[37] Mukherjee M., Bulir D. C., Radford K. (2018). Sputum autoantibodies in patients with severe eosinophilic asthma. *The Journal of Allergy and Clinical Immunology*.

[38] Takiguchi J., Ohira H., Rai T., Abe K., Takahashi A., Sato Y. (2005). Anti-eosinophil peroxidase antibodies detected in patients with primary biliary cirrhosis. *Hepatology Research*.

[39] Smith S. G., Chen R., Kjarsgaard M. (2016). Increased numbers of activated group 2 innate lymphoid cells in the airways of patients with severe asthma and persistent airway eosinophilia. *The Journal of Allergy and Clinical Immunology*.

[40] Sehmi R., Smith S. G., Kjarsgaard M. (2016). Role of local eosinophilopoietic processes in the development of airway eosinophilia in prednisone-dependent severe asthma. *Clinical & Experimental Allergy*.

[41] Mukherjee M., Lim H. F., Thomas S. (2017). Airway autoimmune responses in severe eosinophilic asthma following low-dose Mepolizumab therapy. *Allergy, Asthma & Clinical Immunology*.

[42] Mukherjee M., Paramo F. A., Kjarsgaard M. (2018). Weight-adjusted intravenous reslizumab in severe asthma with inadequate response to fixed-dose subcutaneous mepolizumab. *American Journal of Respiratory and Critical Care Medicine*.

[43] Haldar P., Brightling C. E., Singapuri A. (2014). Outcomes after cessation of mepolizumab therapy in severe eosinophilic asthma: A 12-month follow-up analysis. *The Journal of Allergy and Clinical Immunology*.

[44] Bel E. H., Wenzel S. E., Thompson P. J. (2014). Oral glucocorticoid-sparing effect of mepolizumab in eosinophilic asthma. *The New England Journal of Medicine*.

